# Legal Medicine

**Published:** 1824-03-01

**Authors:** 


### Legal Medicine.

1. *Dr. Castaing*.* Most of our readers must have perused the afflictive trial of Dr. Castaing in the public papers, and have probably made up their minds as to the justice or injustice of the sentence. In the interrogations put to this unfortunate or guilty physician, he admitted that he had made toxicology his study—that he was acquainted with certain vegetable poisons, which left no trace of their action—that he had purchased acetate of morphine to make experiments on animals—that one of the persons deceased had drunk some hot wine in an inn with him—that a servant who tasted the

* London Med. Repos. No. 1, New Series.

wine found it sour—that the deceased had vomited—that he (Castaing) had mixed the acetate with an emetic substance for the purpose of experiments.

Whether the moral or circumstantial evidence was strong, does not appear. The medico-legal evidence was decidedly in Castaing's favour; thus, Laennec deposed, that the deceased might have died of a phthisical complaint—though the symptoms might have been produced by poison—which he was inclined to believe the case. Dr. Michel believed that death was owing to phthisis and not to poison. Dr. Petit thought the symptoms could not have been entirely owing to disease, but *might be* the result of poison. Two apothecaries deposed, that they had sold the prisoner acetate of morphine —and one of them 12 grains of the sulphate of morphine, (by mistake printed sulphate of *soda.*) Orfila deposed, that it was impossible from the *proces verbal* and the appearances, to conclude, that the deceased had died by poison. The appearances on dissection might have been by morphine, but they might also have been the effect of disease. It was proved (or thought so by the jury) that Castaing had forged a will of the deceased. This was strong moral evidence, and we have no doubt that the great strength of the said moral evidence has never reached us; for we are bound to believe, that a jury, (in a case involving no political matters) even in France, would lean to the side of mercy, if they possibly could. He was convicted by a majority of only two—seven against five. It is curious that, in a country where so much importance is attached to legal medicine, no ear appears to have been given to the medical evidence, even of Orfila!—But *all* circumstances considered we fear Castaing was guilty.

---

2. *Forensic Phrenology.* Our readers are aware that Drs. Gall and Spurzheim place the organ of amativeness in the cerebellum, and some insulated facts recorded by authors seem to support the idea of an intimate relation between the brain and the genital organs. The following case related nearly 200 years ago by Hildanus will be read with interest by the phrenologists of the present day.

A consistory court was held at Berne in the year 1630, at which Hildanus and several other physicians assisted, in order to examine Michael Tutzler, 36 years of age, accused of impotency by his wife. Nothing external was defective; but the man himself confessed that eight years previously, he had received a severe blow on his head by a stick, which had deprived him of hearing in the right ear, and had caused for a time, involuntary discharge of urine. From that period "confitebatur *penem erigi non posse.*" Upon this report being made to the court by the medical committee, a divorce was granted.

---

3. *Homicide.** Claudius Noblin and John Jaunet, aged about five or six and twenty years each, had been drinking and carousing

---

* Journal Universel des Sciences Medicales, October, 1823.

in several cabarets, during the 15th November, 1821. About 4 P. M. they quarrelled with two other individuals (a man and his wife) the companions of their debauch. From words it came to blows; and Jaunet received one on the head by a bottle, which prostrated him on the floor. His associate (Noblin) who was, at the moment, engaged in combat with his female antagonist across a narrow table, fell also deprived of sense. This double defeat put an end to the fray, and the spectators hastened to afford assistance to Noblin and Jaunet. The latter, who had been merely stunned by the blow which he received, quickly recovered—but Noblin died on the spot. This circumstance coming to the ears of the police, the body was ordered to be disinterred on the 19th November, two days after inhumation. On the body, there were few or no marks of external violence. The neck was short—the chest large—and much blood had escaped from both nostrils. The conjunctivæ were much injected. On dividing the cranial integuments, great quantities of black blood issued at each cut of the scalpel; and, on removing the skull, the vessels of the meninges, cerebrum, cerebellum, and in fact of the whole encephalon, were found enormously distended with blood. Besides this, there was an extravasation of blood into both lateral ventricles, especially the right—in short, there was here quite enough to account for the death of the patient independently of any external violence. On opening the chest, the lungs were found of a black colour, and extremely gorged with blood. It was thus evident that Noblin died in consequence of a violent determination of blood to the head and chest, accelerated no doubt by the state of inebriety and passion at the moment of the rencontre, and totally independent of any influence from an impression or blow *ab externo*.

Let us now revert to the case of Jaunet. His head presented a contused wound of nearly an inch in extent, accompanied by symptoms of concussion, which, however, were removed by quietude, low diet, and open bowels.

Now suppose Noblin had received this blow from the bottle, instead of Jaunet, what influence would the circumstance have had on the minds of the spectators and of the medical evidence?—It is to be feared that even the legal surgeon or physician would not have been able to divest himself entirely of prejudice on such an occasion. Upon serious reflection, however, it is manifest that the contusion received by Jaunet, would not have altered for worse or for better the fate of Noblin, had the latter received it himself.

4. *Peritonitis resembling Poisoning.** A man 30 years of age, of good constitution, was seized after dinner, on the day before his marriage, with violent colicky pains, and sent immediately for a physician, who noted the following symptoms, viz. pallid countenance, with expression of great suffering—tongue and mouth dry—

* Journal Général Aout, 1823.

thirst—constant nausea—vomiting of liquids tinged with wine—violent pains in the abdomen augmented by pressure, but no intumescence—no evacuations by stool—urine scanty—pulse concentrated. *Twelve leeches to the anus—lavements—diluents*—four ounces of linseed oil that had been taken were vomited up. The next morning brought some alleviation of the symptoms, and the man returned to his own home from the house of his brother, where he had passed the night. On his arrival all the symptoms returned with increased violence, especially the vomiting and abdominal pains. Nevertheless, as every disposition had been arranged for his nuptials, he repaired to the place appointed, and afterwards to the festival common on such occasions. He was soon obliged to retire to bed, and to have emollient injections thrown up. A temporary relief followed; but was soon succeeded by intense fever, and increased vomiting. On the third morning the symptoms were the same, with the addition of abdominal tumefaction—no evacuation by stool—pulse small and concentrated—anxiety extreme—lipothymia. *Ptisans, lavements, semicupia, fomentations.* *4th day.* The vomiting has ceased, but the abdominal pains continue. In the evening, these last became abated, but lipothymia supervened upon the slightest movement. He died on the night of the fourth day.

A death so unexpected, and so soon after a dinner, among his friends, gave rise to strange suspicions, especially as a person had been heard to say, that if he married the woman to whom he was engaged, he would not long survive the marriage. The Commissary of Police therefore gave orders that the body should be examined in a medico-legal manner.

*Dissection.* On opening the abdomen, a considerable effusion of coloured and slightly fetid liquid was found. This was afterwards submitted to proper tests. The omentum was strongly injected, and adhered to the anterior parietes of the abdomen, and, in some places, to the surface of the intestines. The convolutions of the alimentary canal were adherent together in many places, with here and there membraniform flocculi of a purulent appearance. The superior surface of the liver was covered with an albuminous exudation, and the parenchymatous structure was gorged with blood. The internal parietes of the abdomen presented numerous traces of phlogosis, and some purulent exudations, with black spots, produced by congested blood—the mesenteric vessels highly injected. The interior of the œsophagus was natural—the stomach contained a few ounces of glairy and inodorous liquid, which was subjected to test—on its mucous surface, near the cardiac orifice, was a vividly red patch of no great extent—pylorus sound—some patches of red on the internal surface of the duodenum—as also on the mucous membrane of the small intestines, but less and less distinguishable in proportion as the distance from the stomach increased. The large intestines were sound, and no perforations were to be seen in any part of the alimentary canal. The liquids abovementioned exhibited no trace of poisonous impregnation, when submitted to the appropriate tests.

The evidence delivered in was, that the man's death was occasioned by a violent inflammation of the peritoneal covering of the abdominal viscera, without any poison being discovered in the alimentary canal.

"Enfin, ce fait prouve de nouveau la haute importance des remarques que l'on trouve dans les ouvrages de M. M. Foderé, Laisné et Orfila, et desquelles on peut déduire l'aphorisme suivant:—*L'ensemble des symptomes de divers empoisonnemens peut être produit par certaines alterations spontanées des organes digestifs.*"

Our readers will be better satisfied with the medico-legal decision of the physicians than with the medical treatment of the case. A young man seized with abdominal inflammation on the eve of his nuptials, was suffered to die of that inflammation without a vein being opened to check the progress of the disease!

---

5. *Poisoning by Nux Vomica.** A woman, having a quarrel with her husband, swallowed nearly half an ounce of nux vomica. Half an hour afterwards she was found by Mr. O. sitting quite collected and tranquil, her pulse 80. Some little time was lost before an emetic could be procured, which was taken but did not produce effect. In a few minutes she was seized with convulsions, slight and transient at first, but rapidly increasing in force and duration. In one of these she expired, about an hour from the time she swallowed the poison. The vessels of the pia mater were found turgid with blood—an ounce of fluid in the ventricles—heart flaccid and empty —a slightly red patch, nearly half a hand's breadth in size, on the internal coat. No other morbid appearance.

---

6. *Infanticide.*† A woman was tried at a county-town in Scotland for child-murder. She acknowledged concealment of pregnancy. The child's body was found in a press covered with earth. She confessed having placed it there. A piece of twisted calico was drawn twice round the neck, so as to produce a remarkable groove. Another piece was applied tight over the face, and knotted behind the head, so as to flatten the nose. The face was turgid and livid—the conjunctivæ strongly injected with blood. The umbilical cord was divided eight inches from the navel, and had not been tied. No other mark of violence on the body. The lungs were free from putridity, and swam in water, the same as when they are inflated. The air-cells visibly contained air. The examination was eleven days after delivery. The medical witness for the crown deposed, from these appearances, that the child must have been born alive, and that its death must have been caused by suffocation. Three other medical men for the prisoner deposed unanimously, *that if the child had been dead for the period of eleven days, it was impossible for any me-*

---

* Mr. Ollier. Med. Repos. 114.

† Ed. Journal, No. 1, New Series, Jan. 1824.

*dical man to come to a conclusion as to whether the child had been alive at the time of birth.* The woman was accordingly acquitted.

The medical evidence for the crown was, we think, perfectly justified in his deposition. Not so the evidence for the prisoner. There was no putrefaction to account for the lungs swimming in water at so early a period after death. The lungs indeed are peculiarly backward in running into putrefaction, if they have not been used in breathing. We accord with our cotemporary of the North in the severe animadversions which he has made on the conduct of the three medical witnesses above alluded to, who, by the way, had not been present at the opening of the body. The witness for the crown, however, was remiss in not examining whether the child bore marks of hæmorrhage from the untied navel-string. The question, on this subject, seems to have done injury to the crown witness.

